# Theoretical Model for Cellular Shapes Driven by Protrusive and Adhesive Forces

**DOI:** 10.1371/journal.pcbi.1001127

**Published:** 2011-05-05

**Authors:** Doron Kabaso, Roie Shlomovitz, Kathrin Schloen, Theresia Stradal, Nir S. Gov

**Affiliations:** 1 Department of Chemical Physics, The Weizmann Institute of Science, Rehovot, Israel; 2 Helmholtz Center for Infection Research, Braunschweig, Germany; Lehigh University, United States of America

## Abstract

The forces that arise from the actin cytoskeleton play a crucial role in determining the cell shape. These include protrusive forces due to actin polymerization and adhesion to the external matrix. We present here a theoretical model for the cellular shapes resulting from the feedback between the membrane shape and the forces acting on the membrane, mediated by curvature-sensitive membrane complexes of a convex shape. In previous theoretical studies we have investigated the regimes of linear instability where spontaneous formation of cellular protrusions is initiated. Here we calculate the evolution of a two dimensional cell contour beyond the linear regime and determine the final steady-state shapes arising within the model. We find that shapes driven by adhesion or by actin polymerization (lamellipodia) have very different morphologies, as observed in cells. Furthermore, we find that as the strength of the protrusive forces diminish, the system approaches a stabilization of a periodic pattern of protrusions. This result can provide an explanation for a number of puzzling experimental observations regarding cellular shape dependence on the properties of the extra-cellular matrix.

## Author Summary

Cells have highly varied and dynamic shapes, which are determined by internal forces generated by the cytoskeleton. These forces include protrusive forces due to the formation of new internal fibers and forces produced due to attachment of the cell to an external substrate. A long standing challenge is to explain how the myriad components of the cytoskeleton self-organize to form the observed shapes of cells. We present here a theoretical study of the shapes of cells that are driven only by protrusive forces of two types; one is the force due to polymerization of actin filaments which acts as an internal pressure on the membrane, and the second is the force due to adhesion between the membrane and external substrate. The key property is that both forces are localized on the cell membrane by protein complexes that have convex spontaneous curvature. This leads to a positive feedback that destabilizes the uniform cell shape and induces the spontaneous formation of patterns. We compare the resulting patterns to observed cellular shapes and find good agreement, which allows us to explain some of the puzzling dependencies of cell shapes on the properties of the surrounding matrix.

## Introduction

The factors that determine the local and global shape of a cell, are numerous, including the internal state of the cell, with respect to the cell cycle and metabolism, and the properties of the extra-cellular matrix (ECM). Cells that are round while floating in solution, change their shapes dramatically when in contact with a solid substrate [1]–[5]. On a two dimensional surface some cells spread uniformly, while others form elongated extensions (filopodia), or form motile fan-shaped lamellipodia. Inside a three dimensional matrix, cells extend protrusions through their ability to penetrate between the matrix filaments, and by degrading the surrounding material [6]–[8]. These processes have been widely studied in recent years due to the interest in cell motility in normal and cancerous cells, and in relation to the observed dependence of stem-cell differentiation on the properties of the surrounding matrix. Providing a unified model for this large variety of cellular behaviors is difficult, and we aim here to explore the consequences of a relatively simple model, which describes some of the principle forces acting on the cell membrane.

There are several examples of puzzling cellular shape dependencies that have been observed in recent years; (i) Developing neuronal cells have been shown to produce more (less) numerous and shorter (longer) protrusions, when the cells had less (more) actin filament polymerization [9]. (ii) In [7] cells encapsulated in a three-dimensional matrix have been found to have more (less) numerous and shorter (longer) protrusions, when the surrounding gel was stiffer (softer) and therefore harder (easier) to degrade.

While the two examples given above studied the static shapes of cells, there are several studies which investigated the dynamics of cellular shape changes; (i) In [10] the polarization of adhering cells was followed in time, and it was observed that cells initially form numerous and short adhesion “spikes” along the cell perimeter, which later (up to 24hrs) reorganized into two large adhesion regions at the opposite poles of the final elongated cell shape. (ii) In [11] it was observed that cells on a flat substrate, can spontaneously change their shape and cytoskeleton organization between three prototypical forms, which are round (featureless), spiky or ruffled. The cells seemed to randomly switch between these three morphologies over the time course of the experiments.

In recent years, experiments have implicated a large family of curved membrane proteins, for example those containing Bin/Amphiphysin/Rvs (BAR) and IRSp53-Missing-In-Metastasis (IMD) domains, as responsible for sensing (and inducing) concave or convex curvature [12]. Such curved proteins bind preferentially to curved membranes [13]–[15] and the curved domains have also been shown to tubulate membranes [16]. Furthermore, these curved proteins are known to form membrane-bound protein complexes (membrane protein) that include actin-activating components, such as WASP and WAVE [17], [18], and have been found to localize and induce cellular protrusions (filopodia) [19]–[22], and at the leading edge of lamellipodia [23], [24].

In several previous theoretical studies we described how such membrane complexes that have both convex curvature and promote actin polymerization, can induce the spontaneous initiation of membrane protrusions [25], [26]. The intrinsic curvature is an essential since it completes the positive feedback between the membrane shape and local density of membrane proteins; only due to the curvature sensitivity of the proteins do they flow towards the protruding curved parts of the membrane, thereby increasing the cytoskeletal forces acting there on the membrane and leading to the instability and spontaneous formation of protrusions.

Furthermore, it has been shown that the adhesion molecules that connect the cell membrane to the external substrate (such as integrins) aggregate at regions of high convex membrane curvature [5], [6], [27], [28], at the leading edge of motile cells and at cellular protrusions, such as microvilli [29], [30] and filopodia [31].

**Figure 1:**
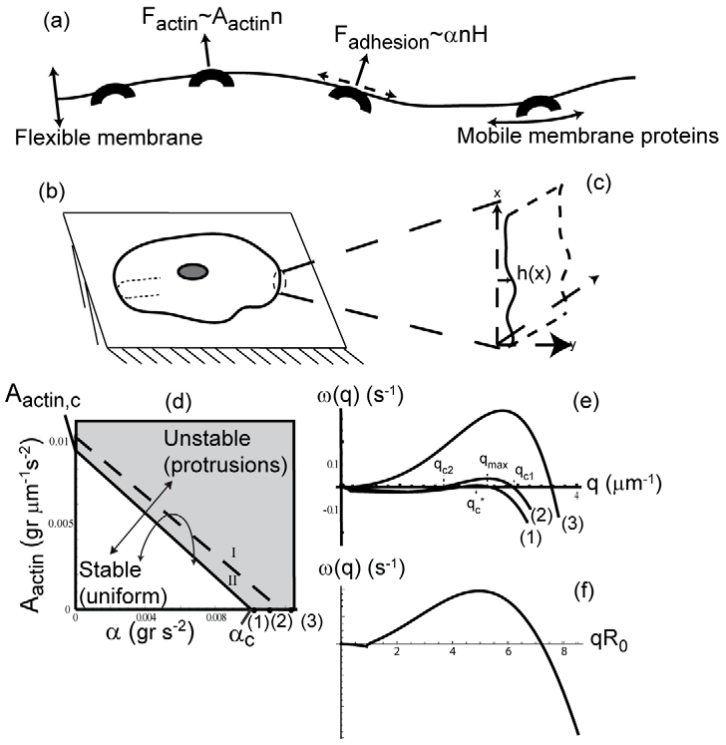
Model scheme and linear stability diagram. (a) Schematic description of the model ingredients: a one dimensional flexible membrane contour, with convex and mobile membrane proteins, which induce normal protrusive forces, due to actin (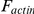) and due to adhesion-driven tension reduction (dashed arrows, 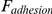). Both forces are linearly proportion to the local membrane protein concentration 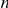, which in our coarse-grained model is treated as a uniform field on length-scales larger than those of the individual proteins. The one-dimensional membrane contour geometries that we calculate (b) round geometry representing the outer contour of a spread cell on a flat substrate, and (c) the flat geometry which describes either a segment of the cell contour or a membrane with translational symmetry. The variable 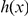 gives the local height deformation of the membrane, relative to its uniform configuration. The curvature at the rim along the cell thickness is indicated by the thin dotted line in (b), and is not considered in our two-dimensional analysis. (d) The phase diagram obtained from the linear stability analysis as a function of the actin protrusive force (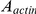) and adhesive strength (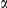). We find two regimes: Stable (uniform) state (below the solid line), and unstable above (gray region, for 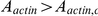 or 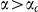). In the unstable regime we find type 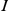 dispersion relation above the dashed line, and type 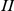 dispersion relation between the solid and dashed lines. The correlation between the actin polymerization and the adhesion strengths is illustrated by two possible trajectories (lines with arrows) in this phase space. (e) The dispersion relations for three different values of 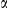 (numbered and marked by bold dots along the 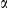 axis in (c)). Negative values of the dispersion corresponds to stable modes, and positive values corresponds to unstable modes. (f) An example of the dispersion relation for the round cell; only the values at integer 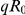 play a role. The real part (solid line) is zero at 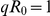 which is an asymmetric mode of translation, while it can be unstable for higher modes.

Thus, we have previously proposed [26] to treat the adhesion molecules as part of the same convex membrane protein that is also responsible for the recruitment of actin polymerization to the membrane (this simplification is discussed further in the Model Details section). The protrusive force in this model can therefore originate either from the reduction in the effective membrane tension due to the adhesion with the extracellular matrix or from the force of actin polymerization (Fig. 1a). For simplicity, we assume that these are the two dominant forces that determine the cell shape; direct contractile forces applied to the membrane are neglected. Also, the role of microtubules (MT) in determining the cell shape is not described here.

Our model does not explicitly describe the dynamics of the cytoskeleton in the cell interior, only close to the membrane. Specifically, the role of contractility induced by myosin-II in the actin network, is not directly accounted for. However, it is possible to effectively take the role of this contractility into account in the present model through the following parameters; (i) The direct contraction of the membrane inwards due to myosin activity, may be included in the values of the effective tension parameter (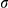) and the effective bulk modulus for the cell's projected area (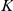). Experiments indicate that both parameters are stiffer when myosin contractility is present, and are softer when it is absent [32]–[34].(ii) In addition, myosin activity is critical for the formation and maturation of adhesion contacts [35]. Therefore the adhesion strength parameter (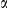) is very much dependent on the myosin activity, again qualitatively. (iii) Myosin activity can furthermore affect the turn-over of the actin and thus modify the rate of actin polymerization, making the parameter (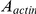) also myosin dependent [36]. While the exact relation between these parameters and the activity of myosin is not known, qualitatively the dependencies should be as described here.

The model presented here is meant to explore the dynamics of cellular shapes driven by the coupling of the cytoskeletal forces with the membrane through curved proteins that can recruit the cytoskeleton activity to the membrane. As a step in this direction, it is therefore important to first understand the behavior of this coupling and feedback, before adding to the model further layers of realism and complexity (which are currently absent). This is the basic philosophy of our approach. The treatment that we present here is general and is not limited to a particular set of curved membrane proteins.

Our model is a coarse-grained model, whereby we do not describe the detailed of the molecular-scale level. The minimal length-scale along the membrane that is relevant to this model is of order 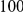 nm. The model is written as a set of equations of motion for the continuum fields that describe the membrane shape and density of membrane proteins, including the actual forces acting on the membrane, and the details of the membrane elasticity.

Other coarse-grained models were recently proposed [37], [38]. These models take a much more detailed description of the actin gel that is pushing the membrane, and the dynamics within the gel away from the membrane itself. Another recent model [39] treats the shape evolution of the cell in terms of a spreading layer of fluid, and relates the instabilities that initiate protrusions to the behavior of such a fluid. These models do not contain however the key component that our model was set up to explore, which is the role of curved membrane proteins that recruit the cytoskeleton forces to the membrane. Other types of models, such as [40], deal with an even more coarse-grained view of the cell. In such models the actual forces acting on the membrane and the membrane elasticity are not explicitly calculated. They are replaced by a kinematic model for the shape evolution, taking into account the biochemical signals that act locally on the cell membrane. These signals represent external and internal pathways that eventually control the cytoskeleton and lead to membrane motion. The huge complexity of the cytoskeleton and membrane dynamics makes it highly beneficial to explore many simplified models, each exploring the consequences of a small set of mechanisms and at different length and time-scales. From the study of these various models we will gain a deeper understanding regarding the many entangled mechanisms that interact within the real cell. It may well be the case that different mechanisms are dominant under different conditions, and therefore control cell morphology under these circumstances.

The model we present here was previously analyzed in the linear limit of small deviations from a uniform flat state [25], [26], which therefore only gave information about the initiation stage of membrane protrusions. In the present paper we explore the dynamics of the protrusions beyond the linear limit, and for closed shapes. Despite the simplicity of this model, which does not describe all the forces that can arise within cells, it may provide a general understanding of the shapes driven by the coupling of the cytoskeleton to the membrane, and shed light on the above mentioned puzzling experimental observations.

## Results

The positive feedback between the protrusive forces (either due to adhesion or actin polymerization), the membrane shape and distribution of convex membrane proteins, leads to a dynamic instability that breaks the uniform configuration and produces membrane undulations where the membrane proteins are aggregated. At the linear regime this was explored in [26]. Our main interest in this work is to follow the evolution of the cell contour shape in two dimensions (Fig. 1b,c), beyond the regime of small perturbations which is captured by the linear stability analysis. In Fig. 1d we plot the stability phase diagram for the system driven by only two types of forces; actin polymerization and adhesion. We wish to explore the long-time evolution and steady-state of the system when it is unstable.

In order to isolate the effects of actin polymerization and adhesion, we used our model to explore along the two axes shown in Fig. 1d, such that we either took 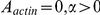 or 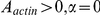. In the real cell these two effects are closely linked, so such a complete separation is done here to better understand the dynamics when each of these factors is dominant. Additionally, in the simulations shown below we limited the amplitude of the membrane undulations to simplify the numerical analysis, but note that the model gives rise to highly elongated protrusions similar to filopodia, when the non-linear membrane tension is set to a small value. Finally, we note that the numerical values of the parameters used in the simulations (see Table 1) were not meant to fit any particular observation, but rather allow us to illustrate the qualitative features of the model.

**Table 1:** List of parameters used in our calculations.

Effective friction coefficient of membrane, 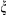 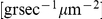	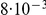
Diffusion coefficient of membrane protein in membrane, 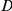 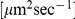	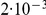
Mean area coverage of membrane protein, 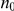	0.1
Saturating density of membrane protein, 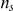 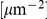	10
Membrane bending rigidity, 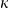 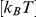	100
Intrinsic membrane protein curvature, 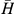 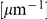	−10
Membrane tension, 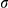 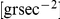	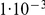
Spring constant, 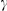 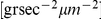	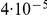
Membrane protein binding interaction, 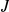 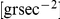	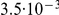
Cell effective bulk modulus, 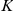 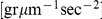	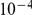
Cell radius, 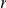 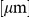	3
Mobility of proteins, 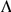	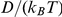
Non-linear tension parameter, 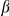 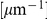	0,0.1,1

### Shape evolution driven by adhesion (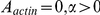)

**Figure 2:**
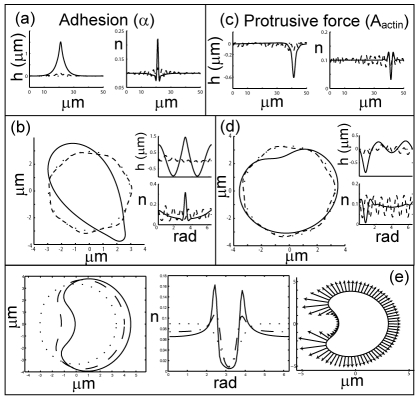
Cellular shapes driven by adhesion and actin protrusive forces. Numerical simulations of the evolution of the membrane shape (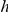 in the flat geometry) and membrane protein distribution (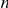), for the flat (a) and round (b) geometries, driven by adhesion only (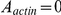): flat- 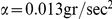, round- 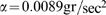. Dotted lines give the initial shape (uniform) and membrane protein distributions uniform with a 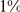 random noise). At an intermediate time equally spaced protrusions form (dashed lines- 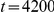 sec), which eventually coalesce to form a single protrusion at the final steady-state (solid lines- 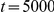). In (c,d) we plot the evolution of the system for the case of only actin protrusive force (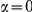), using 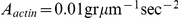. All other conditions are as in (a,b) respectively. We see again equally spaced protrusions at an intermediate time (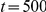 sec), which eventually coalesce to form a single protrusion at the final steady-state (solid lines- 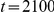
sec). The cell shape in the round geometry was centered at the origin. (e) Evolution of the system driven by actin protrusive force (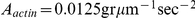), for an initial condition of a highly concentrated Gaussian distribution of the membrane protein. The asymmetric distribution leads at first to a global motion of the cell (dashed line- 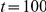 sec), which stops when the steady-state distribution is reached (solid line- 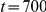 sec). The protrusive forces along the cell perimeter at the steady-state is illustrated by the arrows in the rightmost panel, which are proportional to the local density of membrane protein. The simulations corresponding to (b,d,e) are shown in supporting Videos S1, S2 and S3 respectively.

In Fig. 2a,b we plot the evolution of the system driven by the adhesion forces, for the flat and round geometries respectively. The initial conditions in all cases are those of the uniform equilibrium state (flat or circular respectively), with a random perturbation in the membrane protein density distribution of maximal amplitude 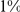. It is immediately clear that the system evolves initially according to the linear analysis, i.e. the most unstable mode from the dispersion relation (Fig. 1e) grows the fastest and the system develops periodic undulations (protrusions) with wavelength 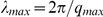.

At longer times the membrane shape and membrane protein density distribution no longer follow the linear behavior, and we observe the coalescence of the protrusions into a single isolated feature. In the flat geometry we end up with a single protrusion which has a sharp tent-like shape, and similarly in the round geometry we find a contour with a droplet shape. The density distribution of the membrane proteins in the steady-state (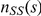), follows very closely the curvature of the membrane (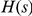). This arises due to the equality between the dominant currents in the steady-state, which are 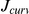 and 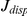 (Eqs.10,11). Equating these currents, we find immediately that: 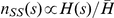 (Eq.S3 in Text S1). The distribution of membrane proteins has a sharp peak at the membrane peak, while depleted everywhere else, due to their convex spontaneous curvature. A further analysis of the steady-state shapes is described in Text S1.

In a real cell the stress-fibers that connect adhesion regions usually impose a bi-polar steady-state with adhesion localized at two opposing poles of the cell. In our model this non-local interaction is absent and therefore the adhesion region can collapse to a single localized domain (Fig. 2a,b).

Note that in the round case, the adhesion forces along the whole membrane are not balanced (their sum does not vanish), since the highly concentrated membrane proteins at the sharp tip give an overall force pointing in that direction (local negative effective membrane tension), Eq.5, and leads to a global drift of the whole cell. This result arises due to the fact that the adhesion-induced protrusive force (Eq.5) depends on the local curvature of the membrane, since it appears as a negative membrane tension term. This means that the sharper the membrane shape at the peak, the stronger is the adhesion force due to both a larger concentration of membrane proteins (larger 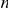 at the peak) and a larger curvature, i.e. the force is proportional to 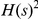. Its integral over the closed contour therefore does not vanish.

### Shape evolution driven by actin polymerization (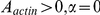)

In Fig. 2c,d we plot the evolution of the system driven by the actin protrusive forces, for the flat and round geometries respectively. The initial conditions are the same as for the case of adhesion-driven shapes (Figs. 2a,b), and similarly the system evolves initially according to the linear analysis, where the most unstable mode from the dispersion relation (Fig. 1e) grows the fastest and the system develops periodic undulations (protrusions) with wavelength 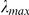.

At later times we again find that the protrusions coalesce, but instead of forming a single sharp peak, the system forms a broad and flat plateau, that is punctuated by a single sharp dip. In the round geometry the membrane develops a broad fan-like bulge, with a smaller concave dip. As for the adhesion-driven system, the density distribution of the membrane proteins again follows the membrane curvature, and is given by 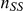 defined above. The membrane protein distribution is therefore rather flat, except for two peaks at the “shoulders” of the membrane dip, and are depleted from the dip itself. A further analysis of the steady-state shapes is described in Text S1.

The protrusive force due to actin on the closed membrane in the round geometry, sums up to zero at the steady-state. This is due to the linear relation between the actin force and the membrane protein density (Eqs.2,3), and that the steady-state membrane protein distribution (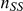) is closely proportional to the curvature, while the integral over the change in the curvature vector vanishes along a closed contour. Until the steady-state shape settles, the forces can be unbalanced, and the whole shape drifts. This is clearly illustrated when we start with initial conditions, where the membrane proteins are localized (in a Gaussian shape) asymmetrically along the membrane contour (Fig. 2e).

Note, that unlike the adhesion-induced protrusive force (Eq.5) whose strength depends on the local curvature of the membrane, the actin protrusive force acts as a local pressure term (Eqs.2,3).

### Dynamics of the approach to the steady-state shape: Coalescence

**Figure 3:**
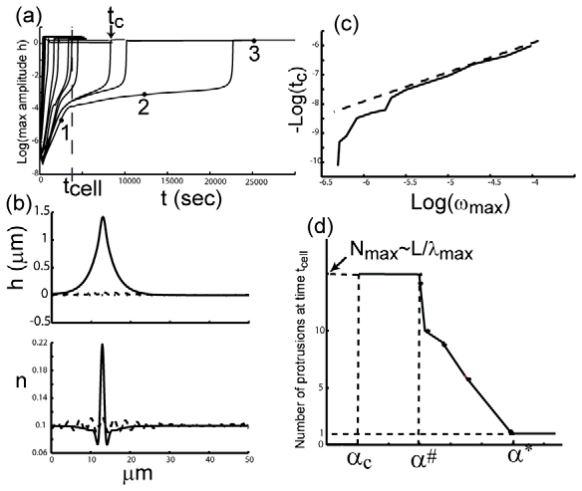
Dynamics of protrusion coalescence. (a) Plot of the maximum membrane amplitude (with respect to the average membrane shape) as a function of time, for decreasing values of the adhesive strength (left to right), approaching the critical values 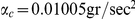 (for 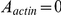, Fig. 1d). The time when a single steady-state protrusion forms is denoted by 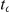, shown for example for one of the trajectories. (b) Typical membrane shapes (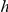) and membrane protein density distributions (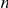) at the marked time points 1-3 in (a). Time point (1) is during the exponential growth of the most unstable mode at wavelength 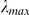 (dotted line), time point (2) is during the stalling in the dynamics as the non-linear tension stabilizes the undulations (dashed line), and time point (3) is at the steady-state of a single collapsed protrusion (solid line). (c) Log-log plot of the observed coalescence time 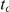 as a function of the maximal value of the dispersion relation 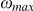. We find a linear relation at large values of 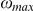 (short 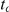, dashed line). A non-linear relation is observed for small values of 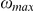, where 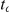 seems to diverge faster. (d) Plot of the total number of protrusions as a function of the adhesion strength 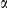, measured at a time representing the cellular time scale 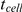 and denoted in (a) by the vertical dashed line (chosen for illustration to be 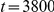 sec). The (arbitrary) threshold for a membrane protrusion to be counted is to be at least 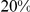 of the maximum amplitude. Above 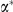 all the simulations reach steady-state before 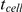 and the total number of protrusions is 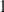. Within the range 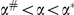 the number of peaks grows with decreasing adhesion strength, till it reaches a maximum number: 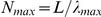. This value then remains unchanged until the critical value of the stability transition 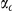, below which there are no protrusions forming and the total number of peaks collapses discontinuously to zero.

In Fig. 3 we plot the evolution of the flat system, driven by adhesion (a similar behavior is observed when actin drives the dynamics), as one approaches the critical point the type-II dispersion vanishes (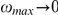) and the system becomes linearly stable (Fig. 1d,e). We observe that initially the amplitude of the fluctuations grow exponentially as: 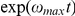 (Fig. 3a). As the amplitude grows, so does the restoring force due to the non-linear tension, which eventually, together with the spring force, stops the growth. The final amplitude 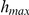 of the steady-state membrane peak (Fig. 3b) is therefore given by the balance between the non-linear tension and the adhesion force of the steady-state shape.

When the coalescence of the protrusions is very slow, close to the instability transition line, non-linear tension is able to stop the growth of the most unstable mode, and the system is stalled with an approximately sinusoidal perturbation (point marked (2) in Fig. 3a). The amplitude of the membrane undulation at this stage is much lower than the final steady-state amplitude, since in the sinusoidal case the membrane proteins are distributed among many peaks, rather than all of them concentrated at one single peak. Eventually small differences among the peak amplitudes and the slow diffusion of membrane proteins break this dead-lock, and allows the further growth of the single steady-state peak. We illustrate these stages in Fig. 3a,b.

In Fig. 3c we plot the dependence of the final coalescence time 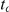 on 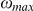, and find a simple linear relation: 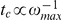, for large 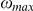. As 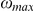 becomes smaller, this relation breaks down due to the partial stabilization of the system in the intermediate state ((2) in Fig. 3a), by the non-linear tension.

### Shape evolution driven by both actin polymerization and adhesion

Although its simpler to analyze the effects of the two active forces separately, as shown above, both are present in a real cell. We now discuss how these two forces act in combination.

Since both adhesion and actin polymerization act to destabilize the membrane, when both forces act together the system is pushed deeper into the unstable regime. This means that the linear instability starts more quickly and with a larger number of protrusions.

**Figure 4:**
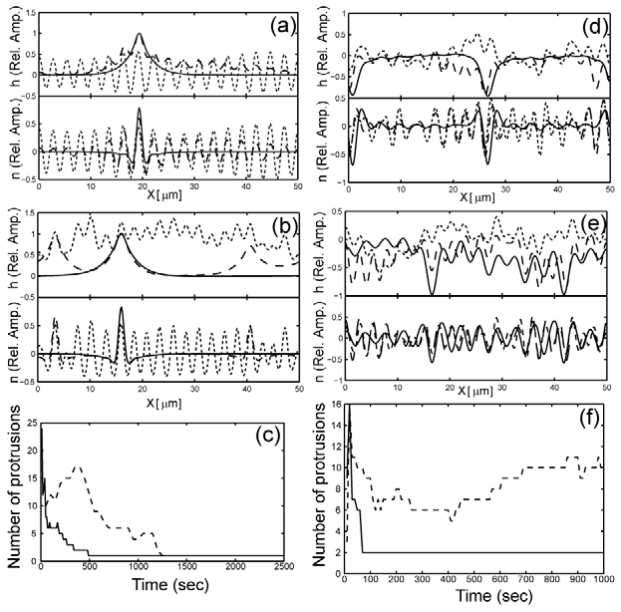
Dynamics driven by both actin polymerization and adhesion. (a,b) Evolution of the flat geometry dominated by adhesion (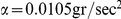), alone (a) and with additional actin polymerization force (b) 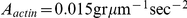. The lines give snap-shots of the system at different times, with the dotted line at the earliest time, dashed line at intermediate time and the solid line at the final time. In (c) we plot the number of protrusions as a function of time for the two simulations (dashed line for pure adhesion, solid line with additional actin polymerization force). (d,e) Evolution of the flat geometry dominated by actin polymerization force (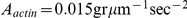), alone (d) and with additional adhesion force (e) 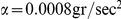. Lines give snap-shots of the system at different times, in the same scheme as in (a,b). In (f) we plot the number of protrusions as a function of time for the two simulations (solid line for pure actin polymerization force, dashed line with additional adhesion force).

However, the long-time non-linear evolution of the protrusions is very different in the adhesion or actin-dominated regimes; in the adhesion-dominated regime, the addition of actin forces results in faster coalescence of the protrusions, as is intuitively expected in a more unstable system. Since the actin acts to broaden the protrusions into fan-shapes, it speeds up local coalescence events. In Fig. 4a,b we plot typical evolutions of the system dominated by adhesion, with and without the addition of actin polymerization. The faster coalescence is shown for these two simulations in Fig. 4c.

However, in the actin-dominated regime we find that additional adhesion forces result in stabilization of the small protrusions that form at the early stages, and suppression of their coalescence. This is due to the fact that the adhesion force stabilizes the pointed tips of the small protrusions, thereby slowing down their broadening and coalescence. This is demonstrated for typical simulations in Fig. 4d-f.

## Discussion

We now wish to compare the results of our model with observations on the shapes of cells. Before we do this we must be aware of the following complication; the membrane shape calculated in our model is dynamically evolving through coalescence of protrusions, and the time-scale for this evolution becomes very long as the critical values of adhesion and actin polymerization are approached (see Fig. 3a,c). A living cell produces local actin or adhesion driven structures over time-scales that vary from several minutes to tens of hours, so when comparing to the calculated shapes we need to be aware that the cellular shapes do not necessarily correspond to the steady-state shapes we predict at very long times. Cells also move around (even adhering cells), and divide and therefore drastically reorganize their cytoskeleton over time-scales that correspond to these two processes. Over such time-scales the cytoskeleton is “reset”, and new features start growing from initial perturbations.

### Cellular shapes

The essential feature of our model is the feedback between the symmetry breaking of the membrane shape and the polarization of the cortical cytoskeleton, i.e. one cannot occur without the other. A recent study [41] demonstrates this property in a cell, where the shape was fixed by an external rigid confinement. It was found that when the confinement imposes a uniform shape, the polarization of the cytoskeleton (excited by an external signal) is transient and decayed rapidly to a uniform state. This observation strengthens the basic mechanism of our model.

**Figure 5:**
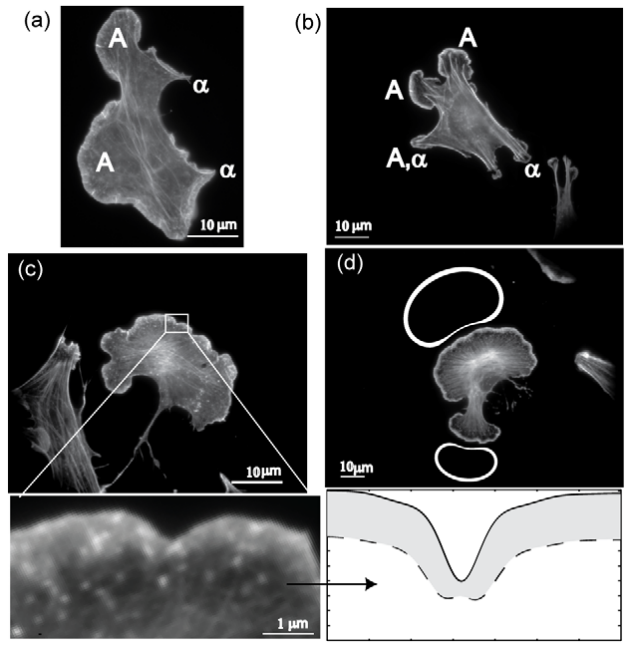
Qualitative comparison between observed and calculated cell shapes. (a,b) Shapes of adhering cells on a flat two-dimensional surface, with fluorescently labeled actin. We denote lamellipodia by “A” and adhesion domains by “
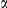
”. (c) A cell dominated by lamellipodia, where we show a segment of the cell perimeter (lower panel) and compare to the calculated (flat geometry) membrane shape (solid line) and cortical actin density (dashed line), assuming a constant rate of actin depolymerization behind the leading edge. (d) Comparison between cell shapes dominated by lamellipodia and the calculated cell shape and cortical actin density (round geometry).

We now compare our calculated steady-state shapes with those of adhering cells on a two-dimensional surface, which are not very motile (Fig. 5). On a qualitative level we see (Fig. 5a,b) that regions of the cell that have strong adhesion (marked by stress fibers) tend to have a pointed tent-like protrusion, as we calculated, while regions that are dominated by lamellipodia-like protrusions (marked by diffuse cortical actin) have a fan-like shape similar to those given by our model. Overlap between adhesion domains and lamellipodia can be seen in Fig. 5b, where adhesion sites seem to serve as platforms for new lamellipodia or vice-versa. Such complex dynamics of overlapping structures is beyond the current version of our simple model.

We can relate the thickness of the observed cortical actin layer along the contour (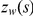) to the local membrane density of membrane protein that we calculate (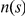), in the following way; assuming that the filaments have a constant rate of severing (depolymerization) 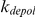 after they are nucleated at the membrane, their number decay as a function of the distance 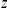 from the membrane according to: 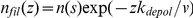, where 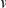 is the treadmilling velocity. The fluorescence signal is proportional to the number of filaments 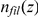, and the images show the signal above some threshold value 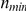, which occurs at a distance: 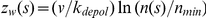. As shown in Fig. 5c,d (using: 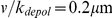 and 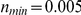) we indeed find that the actin density follows the membrane shape as we calculated; actin is depleted where the membrane has concave curvature.

Another example for the transition in cell shapes from “spiky” (dominated by adhesion points) to “fan-shaped” (dominated by actin polymerization pressure) can be found in [42]. Especially intriguing are the shapes of cells where active Rac1 was expressed, leading to Arp2/3 recruitment to the membrane. These cells were predominantly in a shape similar to those shown in Fig. 2d,e when actin polymerization is the dominant force. In these cells there is a broad fan-shape region, and a single concave depression, exactly as we calculate. It is observed that Arp2/3 is absent from the membrane in the concave region. In our model the actin polymerization is absent from this region due to the local concave curvature, so based on this observation we therefore expect that the Arp2/3 is activated by a membrane-bound complex with convex curvature. Candidates are the WASP-family proteins that have been shown to form complexes with convex proteins [23], [24].

**Figure 6:**
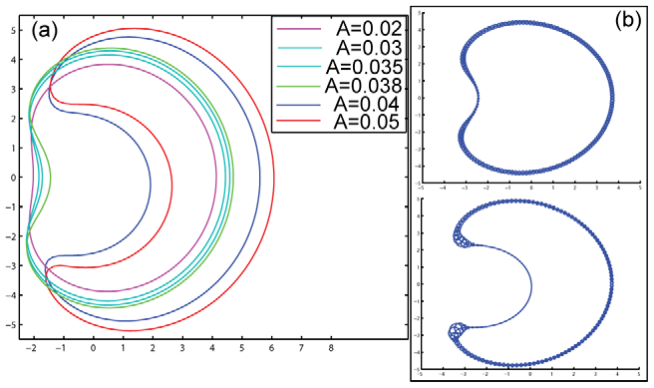
Crescent-cell shape transition at high actin polymerization levels. (a) Calculated steady-state shapes of cells driven by actin polymerization (no adhesion, 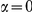), for increasing levels of actin polymerization force parameter: 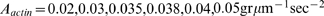 (the non-linear tension parameter was taken to be 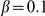). The crescent shapes obtained above a threshold level of actin polymerization are similar to those observed in [34]. (b) Plot of the density distribution of the membrane proteins along the membrane for the two cases of 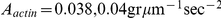 (top and bottom respectively), to demonstrate the strong depletion in the dip region that accompanies the formation of the crescent cell shape. The size of the circles that decorate the cell contour are proportional to the local concentration of the membrane proteins.

Another example for cell shapes that are dominated by actin polymerization comes from the study of spreading cells [34]. In this work it is shown that cells that normally spread in a roughly circular shape, become highly crescent when myosin-II is inhibited by a drug. This effect is attributed to the reduction in the contractile force due to myosin, which allows the protrusive forces of actin polymerization to effectively increase [43] and dramatically alter the cell shape. In our model the myosin activity is not taken explicitly into account, but we can take into account the reduction in contractility by increasing the effective protrusive force of actin (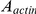 in Eq.2). In Fig. 6 we show that as the actin protrusive force increases there seems to be an abrupt transition in the steady-state cell shape, from a circular shape with a small dip to a crescent-shape cell. In particular we find that there is now essentially complete depletion of the actin nucleators from the membrane in the dip. Our results therefore provide a possible explanation for the dramatic shape transition reported in [34] when myosin-II was inhibited. Specifically, it was noted in [34] that there is complete absence of branched actin polymerization near the membrane in the dip region, as we find in our model.

Finally, an example that comes from motile cells is given in [44], where it was observed that the localization of Ena/VASP to the leading edge is responsible for the formation of a cell with a single fan-shape (as we find in Fig. 2d,e). When the Ena/VASP was not localized to the edge, the cell assumed a more round and fluctuating shape. We note that Ena/VASP can be localized to the membrane by association with IRSp53 [45], which is precisely the type of linkage that our model proposes. In addition, when the cell membrane was forced to have a flat edge, it was observed that the localization of the Ena/VASP in this region disappeared, and reappeared only when the constraint was removed. This observation again points to the role of membrane shape in the localization of this protein at the leading edge, as we propose.

### Evolution of cellular protrusions: Coalescence

While the coalescence phenomenon was previously discussed theoretically in [46], [47], our work is the first to our knowledge, to calculate this process explicitly for actin and adhesion driven protrusions on a membrane. The coalescence dynamics of the membrane protrusions that we calculate (Fig. 3), allows us to propose an explanation for a number of long-standing puzzles regarding the relation between cellular shapes and the properties of the surrounding matrix. Our model predicts that initially the adhesion or actin-driven membrane undulations grow at the wavelength 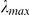, so that the number of cellular protrusions along a membrane contour of length 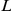 is of order 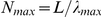. The system then evolves by coalescence of the protrusions into progressively fewer and larger protrusions, until a single feature remains (Figs. 2,3b).

It is hard to find much published data to compare with this general feature of our model. This type of dynamics was observed for adhering cells in [10], where the adhesion foci along the cell rim were initially numerous (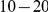 after 4 hrs) and spread at rather uniform spacing, but later formed roughly two adhesion regions at the two opposing poles of the elongated cell (after 24 hrs). Our model qualitatively captures these dynamics (Fig. 2b), although it does not lead to a bi-polar distribution since we do not have the constraint imposed by the need to connect opposing adhesion regions by internal stress-fibers. Recently, the dynamics of cell morphology during spreading and adhesion was more closely investigated experimentally [39]. In this work one can observe some of the shape evolution we calculate, such as the formation of regularly spaced protrusions from an initially circular cell (Fig. 1 of [39]), as well as the later coalescence of such structures (Fig. 5 of [39]).

Furthermore, our model predicts that as the phase transition line is approached (Fig. 1d) the time-scale for coalescence of the protrusions becomes very long (Fig. 3a,c). Since the cell has a typical time-scale (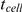) over which it reorganizes its cytoskeleton (determined by external cues or division time, etc.), it is relevant to compare the calculated shapes at this particular time. We plot in Fig. 3d the number of protrusions at this chosen time and find that it has the following non-linear behavior; for values of the adhesion or actin forces (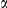 or 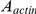) that are below the critical threshold, the system is stable (uniform) and the number of protrusions is therefore zero. Just above the critical line (either 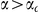 or 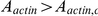, Fig. 1d) the time-scale for coalescence (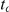, Fig. 3c) is much longer than 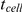 and the observed number of protrusions is simply the maximal one 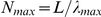 (note that near the transition 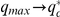, Fig. 1e). As 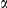 (or 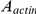) increase further the coalescence time becomes shorter and the protrusions begin to coalesce by the time 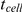, consequently reducing the number of protrusions we count. Above a certain value of the adhesion or actin parameters (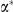 or 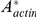), we arrive in a regime where: 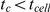, and the protrusions have all coalesced to form a single feature. We count a membrane protrusion if its amplitude is at least 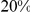 that of the largest membrane protrusion. This value of the threshold was chosen arbitrarily, but does not change the qualitative behavior.

This unique prediction from our model suggests a possible explanation for the following puzzling observations; (i) In [9] neuronal cells adhering to a flat two-dimensional surface, have been shown to produce more (less) numerous and shorter (longer) protrusions, when the cells had less (more) activity of actin filament polymerization. This observation may correspond in our model to the cells having their actin force parameter vary within the region: 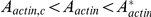. Note that the number of protrusions is stabilized (i.e. further coalescence is suppressed) in these cells when MTs invade the nascent protrusions along the cell edge. This invasion process sets the time scale 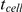. (ii) In [7], cells encapsulated in a three-dimensional matrix have been found to have more (less) numerous and shorter (longer) protrusions, when the surrounding gel was stiffer (softer) and therefore harder (easier) to degrade. We can map the ability of cells to degrade their surrounding matrix and protrude with the parameter describing the actin protrusion force, such that a stiffer gel corresponds to a smaller 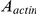, and vice versa. The observations therefore suggest that the regime of stiffness explored in the experiments corresponds again to: 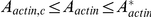. Note that in this experiment it was reported that when the stiffness increased above some threshold the number of protrusions collapsed to zero, as our model predicts (Fig. 3d). A similar relation between the number of degradation-protrusions and substrate stiffness was also observed in [48], in the context of invadopodia produced by cancer cells during invasion of the ECM.

Most recently the number of cellular protrusions (“fingers”) was measured as a function of the actin polymerization activity, using a drug [39]. As our model predicts (Fig. 3d), the measurement shows that as the actin polymerization is inhibited the number of protrusions increases.

The effects of the adhesion strength of a cell on a flat two-dimensional surface, on the cell shape have been explored in a number of papers [1], [49]–[51]. As the stiffness of the substrate is increased, so does the strength of the cell adhesion, and our model would therefore predict a decrease in the number of protrusions and more global cell polarization, as the stiffness increases. Indeed this is the observed trend.

The above discussion suggests that cells seem to naturally live in a parameter space near the transition lines between the stable (uniform) and unstable (protrusions) regime (Fig. 1d). Such a location may allow cells to change their shape by only small changes to their cytoskeleton activity. This feature may explain the spontaneous cellular transitions observed in [11], from uniform round cells to cells covered by filopodia.

Note that there are several effects that may strongly suppress or delay the process of protrusions coalescence; long actin-driven protrusions, such as filopodia, can get anchored to the external substrate at their tips, and stabilize in such a way that any coalescence with neighboring filopodia is suppressed. We demonstrate such dynamics in our model, where a small addition of adhesion stabilizes the actin-driven protrusions (Fig. 4d-f). Additionally, strong adhesion may decrease the effective mobility and diffusion coefficient of the membrane proteins, again delaying or suppressing coalescence. This second process was not explicitly treated in the current model, but can be added in the future. These processes may result in cells retaining their “polygonal” (or “spiky”) morphology, where by protrusions are separated by the typical wavelength 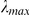.

Finally, we have not discussed here the processes that degrade cytoskeleton-membrane structures (protrusions and adhesion complexes). While in our model the overall number and activity of the membrane proteins is constant, in the cell each protein undergoes processes of degradation and deactivation. Such processes endow each cytoskeleton-membrane structure with a finite lifetime [52], which increases with the size of the protrusion and its protein content. The degradation process therefore further acts to inhibit coalescence as we approach the critical transition line where coalescence slows down (Fig. 3). When the coalescence process is faster than the degradation, we will find cells that reach the steady-state shapes and attain global polarization, while slow coalescence will be further inhibited by the decay of the protrusions and the initiation of new ones (see for example [53]).

### Cell motility

Although we have been interested in cell shapes rather than cell motility, there are two features of our model that may be relevant for this problem as well, and suggests that curvature-driven feedback may play a role in cell motility as well.

1. In our model of the round cell driven only by adhesion (Fig. 2b) there is spontaneous symmetry breaking due to the feedback between the convex membrane protein and their induced protrusive force. The protrusive force is driven by a local negative membrane tension which amounts to the continuous addition of membrane area at that location. Along the rest of the cell, the positive membrane tension pulls the cell rear inwards, and therefore an overall drift in the direction of the sharp feature ensues. Our model may therefore be relevant for the study of amoeba-type cell motility observed by cells in a three-dimensional matrix [8], where localized adhesion to the matrix at the leading edge is the dominant feature, and cells often have the tear-drop shape we calculate.

2. In our model of the round cell driven only by actin protrusion (Fig. 2d) there is overall symmetry breaking and the shape is very similar to that observed in highly motile cells, such as keratocytes [54], moving on a two-dimensional surface. Within our model there is a global cancelation of the protrusive forces such that the cell is stationary, and this cancelation arises due to the strong backward forces at the two highly curved corners, where the membrane shape is most convex (Fig. 2e). These backward forces cancel the forward protrusive force along the fan-like front of the cell. In a real motile cell the actin seems to be prevented from pushing effectively the membrane backward at the cell back, due to myosin activity and polarization of the actin depolymerization processes [55], [56]. Our model suggests that the localization and shape of the leading edge may be maintained by curved activators of actin [23], while additional symmetry breaking processes, such as retrograde flow and maturation of the adhesion contacts, are necessary to polarize the actin polymerization and result in overall cell motility [57]–[59].

### Conclusions

To conclude, let us summarize our main findings:

- When protrusive forces, due to either actin polymerization and/or adhesion, are recruited by convex membrane proteins and exceed a threshold value, we find that protrusions spontaneously form, initially at regular spatial intervals. The protrusions evolve by a process of coalescence, leading to larger but fewer protrusions with time.
- The shape of the protrusions at long times differs significantly between the cases dominated by adhesion (pointed tent-like) or actin polymerization (broad fan-shaped), as observed in cells.
- The time-scale for the coalescence of protrusions diverges as the critical threshold of cytoskeleton activity is approached (from above). This means that the observed number of protrusions increases near the threshold value (from above) and vanishes below it. This type of dynamics can explain the puzzling observed dependencies of cell shapes on the properties of the surrounding matrix [7], [9].

Note that the process of pattern coarsening, which in our case is in the form of coalescence of protrusions, is a more general phenomenon than our specific model, and it appears in other biological systems as well [60]. Therefore our conclusions regarding the interplay between the cellular time-scales and observed patterns are also more general and may apply even if the underlying mechanisms for protrusion formation are different from those considered here.

Our model not only recapitulates many features of observed cellular shapes, but also allows us to make predictions that await further measurements. It highlights the major role of curved membrane proteins that couple the membrane to the underlying cytoskeleton in determining cellular shapes [12], [23].

Let us emphasize again that the work presented here is just one step towards the understanding of the coupling of the cytoskeleton to the cell membrane. Our philosophy is to start with a simple model, which does not include all the complexity of the cell. We believe that the results we obtained are interesting and rich enough to encourage future extensions of our model, that will indeed add more details.

## Materials and Methods

### Experimental methods

Murine fibroblastoid cells were as described before [61], [62]. Cells were grown in DMEM, 4.5 g/L glucose (Invitrogen, Karlsruhe, Germany) with 10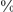 FCS (Sigma-Aldrich, Munich Germany), 2 mM glutamine, 1 mM sodium-pyruvate, 0.1 mM non-essential amino acids (Invitrogen) at 37°C and 7.5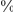 CO_2_. For visualization of the actin cytoskeleton cells were grown on acid-washed glass-coverslips. For the induction of lamellipodia cells were seeded sub-confluently onto glass-coverslips, serum-starved over night, and treated with DMEM alone or DMEM containing 10 ng/ml PDGF-BB (PDGF-BB; Sigma-Aldrich) for 5 min prior to fixation. Cells were then fixed with formaldehyde (4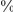) in PBS for 20 min, extracted with 0.1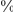 Triton X-100 in 4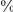 PFA for 1 min, and stained with Alexa dye-labelled phalloidin (Invitrogen) as described. Samples were analyzed on an inverted microscope (Axiovert 100TV; Zeiss, Jena Germany) using a 63x/1.4-numerical aperture plan-apochromatic objective and equipped for epifluorescence as described previously [63]. Images were acquired with a back-illuminated, cooled charge-coupled-device camera (TE-CCD 800PB; Princeton Scientific Instruments, Princeton, NJ, USA) driven by IPLab software (Scanalytics Inc., Fairfax, VA, USA). All microscopic images were further processed with Adobe Photoshop 7.0/CS software (Adobe Systems, Mountain View, CA, USA).

### Model details

In this section we describe the theoretical model that we used. We start with the derivation of the basic equations of motion for the membrane shape and density of membrane proteins. We then describe the linear stability analysis of the flat and round membrane shapes.

The geometries which we will explore in this work are only two-dimensional. In three dimensions there is the additional degree of freedom of the membrane proteins to re-orient themselves in response to changes in the local curvature tensor [64]. Their dynamics and distribution along the membrane will therefore be modified, and may well affect the long-time evolution of the membrane protrusions, while the short-time linear regime will not be very much affected by the dimensionality.

The first geometry is that of a round closed shape (Fig. 1b), which can describe the outer contour of a flat cell that is adhered on a substrate. Such a geometry arises in many experiments where cells spread over a solid surface. In this geometry we assume that the cell has a preferred overall projected area (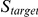), which it tries to maintain while its shape is evolving [38].

The second geometry describes a segment of the cell outer contour, where the membrane is initially straight (Fig. 1c). This geometry can also describe a flat two-dimensional membrane, under the constraint of having undulations with translational symmetry. In this geometry we need to pin the membrane using an external harmonic force (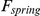, Eq.6), to prevent its drifting motion. Such an external force mimics the effects due to the adhesion of the rest of the cell to an external substrate.

Note that we do not explicitly describe the membrane shape along the cell thickness (Fig. 1b). If the membrane curvature along the thickness is roughly constant, then it simply enters our calculation as a modified membrane tension and adhesion strength, as well as changing the value of 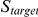 in Eq. 2 (see more details in Text S1).

Regarding the membrane proteins in our model, we assume that their overall number on the membrane is conserved, and that they are allowed to dynamically move along the fluid membrane.

In our model the adhesion and actin protrusive forces are described by two independent parameters [26] (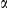 and 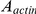, Fig. 1d). In the cell the actin polymerization activity and adhesion are closely related [65], [66]; actin polymerization and treadmilling induces the initiation of focal adhesions [67], so the adhesion strength (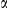) increases with the actin polymerization activity (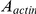). On the other hand, the protrusive force on the membrane due to actin polymerization is found to depend in a biphasic manner on the adhesion strength [1], [2], [4]; it increases for low adhesions as the traction with the substrate increases, but eventually decreases as strong adhesion stalls the membrane. These two types of dependencies are indicated by the trajectories drawn in Fig. 1d. Furthermore, the actin organization is different where adhesion or polymerization dominate; mature adhesion sites have internal actin stress fibers that have little direct contact with the membrane, while regions dominated by actin polymerization, such as filopodia and lamelipodia, have actin filaments that push actively the membrane surface. These two different scenarios lead us to describe these two forces by two independent parameters 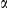 and 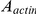.

Inside cells the actin polymerization rate should also depend on the local membrane restoring force applied to the growing tips [68]. This relation is not well understood inside the cell, where the mechanism for polymerization depends on the membrane composition and type of actin nucleator. We can implement this effect in our model by making the polymerization rate 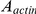 dependent on the membrane force, when this force opposes the actin protrusion [69]. This effect does not modify the (linear) stability of the system, but does change the shapes and dynamics of the resulting membrane undulations. In Text S1 we give an example of a calculation where we demonstrate the effects that this adds (Fig. S1). For simplicity we use a constant (uniform) 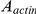 for the rest of this paper. Note that it is easy to implement within our model a non-uniform actin polymerization rate or adhesion strength (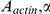) by making these parameters dependent on the local density of membrane proteins, membrane shape etc.

The actin polymerization in our model produces a force that is acting normally on the membrane, similar to an internal pressure force (see Eq.2). This may be a good description for Arp2/3-induced actin polymerization where a rather uniform network of filaments, with a distribution of angles, is protruding against the membrane surface. Actin polymerization that is induced by Formin-type proteins tends to be unidirectional [63], and the actin-membrane interactions of the resulting actin bundle can be strongly influenced by binding proteins and molecular motors [70], [71]. These additional effects are not explicitly treated here, and could be added in the future.

Another important point to note regarding our model, is that we treat the adhesion as a localized event on the membrane surface, while in adhering cells mature adhesion sites require stress-fibers that link adhesion domains on two distant locations on the membrane [65]. This non-local feature of adhesion is absent from the present model.

### Equations of motion

Our model investigates the coupling between both the adhesion and the actin protrusion forces to the membrane curvature. We give below the free energy expression used in the model, from which we derive the equations of motion of the membrane shape and density distribution.

The continuum free energy for the model is based on the Helfrich form [72], including the membrane proteins interactions and entropy [26].
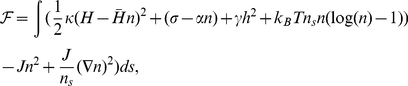
(1)where 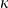 is the membrane bending rigidity, 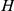 is the local mean membrane curvature, 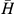 the intrinsic curvature of the membrane protein, 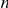 is the fractional area coverage of the membrane by the membrane proteins, 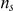 is the saturation density of membrane proteins on the membrane, 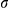 is the membrane tension, 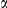 is a proportionality constant describing the effective adhesion interaction between the membrane proteins and the external substrate, 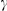 is a restoring spring term, 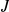 is the direct binding interaction energy between the membrane proteins, and 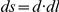 is an element of membrane area, where 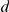 is the thickness of membrane represented by our contour and 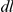 is a line element along the membrane contour.

The first term in Eq.1 gives the curvature energy due to the mismatch between the membrane curvature and the spontaneous curvature of the membrane protein. The second term describes the negative contribution to the effective membrane tension, induced by the adhesion molecules. The third term describes an external harmonic potential that pins the membrane (in the flat geometry), representing the overall localization of the cell to the external matrix. The fourth term gives the entropic contribution due to the thermal motion of the membrane protein in the membrane. The fifth and sixth terms are the bulk and surface aggregation energies of the membrane protein.

Note that for simplicity in our model we have a single species of membrane protein complexes, described by the field 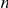, which has the ability to both recruit actin polymerization and/or adhesion. In reality these two properties may exist on two (or more) independent membrane complexes, with different curvatures and interactions (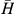 and 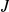 in Eq.1). Such an increased level of detail, and complexity, can be introduced in future elaborations of this model.

We next derive the equations of motions for a general contour in two dimensions, using the variation of the free energy (Eq.1) with respect to the membrane coordinate [73], [74] and membrane protein concentration [74], and adding the active forces due to actin polymerization which cannot be derived from the free energy [25], [26]. To take into account the drag force on the cell membrane due to viscous forces, we assume for simplicity only local friction forces [26], [74], with overall coefficient 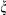. Note that the local friction coefficient for membrane motion contains also the effects of adhesion [26], so that: 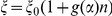, where 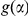 is some increasing function of the adhesion strength 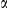, representing the stick-slip nature of the adhesive bonds [75], [76]. This term leads to non-linear effects, which do not modify the (linear) stability of the system or its qualitative dynamics, simply slows them down.

Before we give the equations of motion let us note again that we are interested in two geometries of the membrane; one is a closed, round shape which describes a whole cell, while the second is a flat membrane that describes a segment of the entire cell. For the round shape the equation of motion of the contour coordinate, 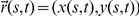, is given by
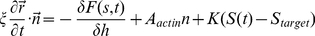
(2)where 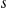 is the index along the contour length, 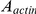 is the proportionality factor representing the actin protrusive activity induced by the membrane proteins, 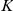 is an effective bulk modulus for the cell's projected area, 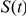 is the area enclosed by the contour, and 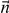 is a unit vector normal to the contour. The variation of the free energy is projected to give the forces normal to the membrane contour [73], [74]. The protrusive force of the actin is assumed to grow linearly with the local concentration of membrane protein [25], [26] and the area-preserving forces act as a global internal pressure. Both of these forces act normal to the membrane. Note that in this geometry we do not use the spring term in the free energy (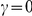 in Eq.1).

For the flat geometry the equation of motion of the membrane height 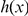 is given by
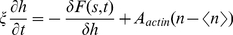
(3)where we subtract the average actin force to prevent the membrane drift, and 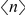 is the average fractional area coverage of the membrane proteins along the membrane contour. Additionally, in the free energy we use a non-zero spring term to prevent an overall drift of the membrane. In this geometry we only take the projection of the force along the 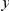-axis, since the position of the membrane along the 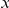-axis is fixed.

We now list the forces (per unit area) derived from the variation of the free energy (Eq.1) [73], [74]

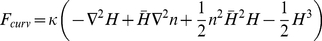
(4)

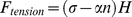
(5)

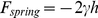
(6)

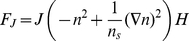
(7)where 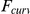 is the force due to the curvature energy mismatch between the membrane curvature and the spontaneous curvature of the membrane proteins, 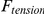 is the membrane tension force, 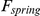 is the harmonic pinning force and 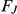 is the force due to the aggregation potential of the membrane proteins. There is in addition a force arising from the entropy of the membrane proteins in the membrane, which acts to expand the length of the contour, and has the form: 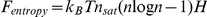. We have neglected this force in our calculations, since it is smaller than the other forces. All the derivatives are along the contour length (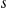).

In a cell the membrane area is finite and this leads to a non-linear form for the effective membrane tension [77]

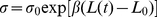
(8)where 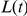 is the contour length, 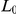 is the initial contour length and 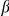 is the factor that determines the length-scale at which the non-linear growth in the tension sets in. This restraint on the amplitude of membrane undulations also allowed us to avoid kinetically trapped configurations, by preventing the strong depletion of the membrane protein density which would have slowed down the evolution of the system, since it depends on the currents of membrane protein flowing in the membrane. Regions where the membrane protein density is highly depleted act as effective barriers for such flows [60]. A previous study [60] suggests that the steady-state of the system is unaffected by this limitation, only the rate at which the system is able to approach this steady-state.

We now calculate the dynamics of the membrane protein density, using the following conservation equation (covariant version [78]) and the free energy 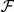 [79] (Eq.1)
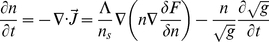
(9)where 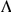 is the mobility of the proteins in the membrane and 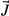 is the total current of membrane proteins on the membrane, which includes the following terms
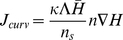
(10)

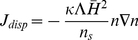
(11)

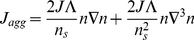
(12)

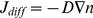
(13)where 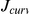 is the flux resulting from the interaction between the membrane proteins through the membrane curvature, 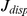 is the dispersion flux due to the membrane resistance to membrane protein aggregation due to their membrane bending effects, 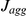 is the flux due to the direct membrane protein aggregation interactions, and 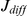 is the usual thermal diffusion flux, which depends on the diffusion coefficient, 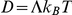.

The last term in Eq.(9) arises from the covariant derivative of the density with time on a contour whose length evolves with time [78]. In this term 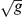 is the matrix tensor, which in our one dimensional contour is simply the line element 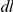. This term ensures that the total number of membrane protein is conserved as the contour length changes.

Note that we have used here a constant value for the mobility of the membrane proteins (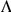), but this mobility is in reality diminished with increasing adhesion strength 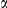. Furthermore, crowding effects in the membrane decrease the mobility with increasing local concentration of membrane proteins 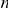. We checked that both of these effects do not qualitatively change the results that we present in this paper, where for simplicity we take 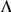 (and therefore also 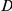) to be a constant, independent of 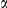, 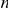 or the local shape of the membrane [80].

### Linear stability analysis

We next performed a linear stability analysis of the model, as was previously done for the flat case [26], in order to find the regions of instability of the system, and for the round case to calculate the equilibrium radius and membrane protein density. Note that we will consider only convex shape for the membrane proteins (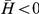), in order to get instabilities in the dynamics of this system [25], [26].

#### Flat geometry

In the flat geometry the contour is allowed to evolve only along one direction and we label the amplitude of the membrane height fluctuation as 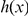 (Monge representation), where 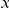 is the coordinate along the initial contour length. In this representation the linearized curvature is: 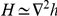, and the length element of the contour 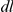 is given by: 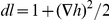. Linearizing the equations of motion (Eqs.3–9), we then Fourier transform to get a 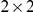 matrix whose eigenvalues 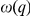 give the dynamic evolution of small fluctuations from the equilibrium uniform state. Both eigenvalues are real, and one of them is always negative and therefore represents only damped modes. The second solution can become positive in some a range of wavevectors and for certain parameters of the model, representing unstable modes that grow with time. The parameters of the model that represent the effects of actin polymerization and of adhesion are 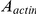 and 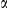 (Eqs.2,3,5).

Fixing all the other parameters, we plot in Fig. 1d the phase-diagram from the linear stability analysis as a function of 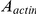 and 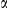 [26], where the three numbered points correspond to the adhesion strength used to calculate the dispersion relations given in Fig. 1e. Below the solid line in Fig. 1d the system is linearly stable and uniform, while above this line there are unstable modes and spontaneous patterns are initiated. The instability is driven by the positive feedback between the membrane shape and the density of membrane proteins [25], [26], due to their induction of protrusive forces and convex spontaneous curvature. Between the solid and dashed lines there is a growing range of wavevectors, 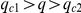, which are unstable (Type-II). Above the dashed line this range extends to zero wavevectors (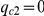, Type-I). The most unstable mode (largest positive 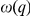) is denoted by wavevector 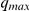. Along the solid line, the type-II instability first appears at a wavevector 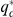, which increases as 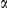 (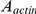) increases (decreases).

#### Round cell geometry

For the round geometry, we follow a similar linear stability analysis, where we replace the curvature by the following expansion (as a function of the angle 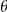)
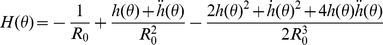
(14)where 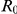 is the initial radius and 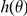 is the deformation of the membrane in the radial direction, and the differentiation is with respect to 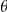. The line element 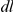, is also expanded up to quadratic order
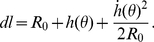
(15)


The differences compared to the flat geometry is that we do not include now a spring energy term, and the actin protrusive force does not have the mean force subtracted. Note also that the area-conserving term appearing in Eq.(2) does not contribute to the linear stability analysis. Following the same methods as described for the flat shape membrane, we linearized the equations of motion, and solved the dispersion relation. In this geometry there is a uniform force acting on the membrane, which vanishes for the initial equilibrium circular shape. This condition determines the initial radius 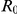 and the initial uniform membrane protein area coverage which is defined by: 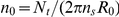, where 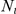 is the total number of membrane proteins on the initial contour. The dispersion relations for the circular geometry are very similar to those shown for the flat case in Fig. 1e, while the wavevector 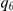 has only integer values.

### Numerical simulations

The main results of our work are calculated using numerical simulations of the dynamics of our model system beyond the linear limit. For this purpose we solved Eqs.(2–9) using an explicit Euler method in Matlab. We checked for the convergence of our one-dimensional simulations, in space and time. We used occasional re-discretization of the contour into equally spaced nodes, using the cubic “spline” routine, to prevent large changes in discretization density along the contour as it evolves with time. After such operation we re-distributed the membrane protein density among the new node locations using a linear interpolation algorithm, such that the total membrane protein number is conserved.

In the flat geometry we used the Monge representation 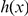, which leads to a complex curvature restoring force (Eq.S6, Text S1), but in order to simplify the numerics we eventually kept the curvature force only up to linear order. The boundary conditions on the flat geometry were taken to be periodic, for simplicity. In the this geometry, the membrane moves only along the direction perpendicular to the initial flat state.

## Supporting Information

Figure S1 (a) Numerical simulations of the evolution of the membrane shape and membrane protein distribution, for the flat geometry, driven by actin alone. Black lines give the results without the effects of the local force, while red lines are including the effects of the local membrane restoring force. (b) The number of protrusions as a function of time for the two calculations shown in (a), with the same color code.
(0.78 MB EPS)

Text S1 Cellular shape transitions: Supplementary Information section. PDF file containing the supplementary information, and movie captions.
(0.05 MB PDF)

Video S1 Movie of a simulation showing the cell shapes evolving due to adhesion, corresponding to Fig. 2b.
(0.67 MB AVI)

Video S2 Movie of a simulation showing the evolution of a polarized cell shape due to actin polymerization, corresponding to Fig. 2e.
(0.51 MB AVI)

Video S3 Cell shape driven by actin polymerization. Movie of a simulation showing the cell shapes evolving due to actin polymerization, corresponding to Fig. 2d.
(1.63 MB AVI)
