# Supplementary material for: Theoretical Model for Cellular Shapes Driven by Protrusive and Adhesive Forces
Source: PLoS Comput Biol. 2011 May 5;7(5):e1001127. doi: 10.1371/journal.pcbi.1001127 (PMC3088653; doi:10.1371/journal.pcbi.1001127)
Supplement: Text S1 — Cellular shape transitions: Supplementary Information section. PDF file containing the supplementary information, and movie captions. (0.05 MB PDF) [file pcbi.1001127.s002.pdf]

Theoretical model for cellular shapes driven by protrusive and adhesive forces: Supplementary Information section

Doron Kabaso[1], Roie Shlomovitz[1], Kathrin Schloen[2],
Theresia Stradal[2] and Nir S. Gov[1,*]
1 Department of Chemical Physics,
The Weizmann Institute of Science,
Rehovot 76100, Israel
2 Helmholtz Center for Infection Research,
Inhoffenstraße 7, 38124 Braunschweig,
Germany

1 Analysis of the the SS solution for the flat geometry

We wish here to derive an approximate steady state solution near the extrema of the shape (peak or minimum), in order to shed light on the underlying mechanisms responsible for the observed steady state shapes (Figs.2a,c). In the steady state, the sum of fluxes of membrane-bound protein complexes (membrane proteins) is equal to zero. In particular, we found that in our calculations the dominant fluxes are the flux due to the membrane intrinsic curvature, J_{curv}, the dispersion flux, J_{disp} and the first term in the aggregation flux J_{agg}. Equating these terms gives

$$\frac{\kappa\Lambda\bar{H}}{n_s}\left(\nabla H - \bar{H}\left(1 - \frac{2J}{\kappa\bar{H}^2}\right)\nabla n\right) = 0. \quad \text{(S1)}$$

The result is

$$\nabla n = \frac{1}{\Gamma\bar{H}}\nabla H, \quad \text{(S2)}$$

where $\Gamma = (1 - 2J/\kappa\bar{H}^2)$. By integrating both sides of Eq.S2, we get

$$n(s) = n(0) + \frac{H - H(0)}{\Gamma\bar{H}}. \quad \text{(S3)}$$

The function representing the membrane shape near the peak (or minimum) region at the steady state is approximated by a fourth order polynomial. Due to symmetry around the peak the odd orders of the polynomial are zero. We use the proportionality found in Eq.S3 to obtain the distribution of membrane proteins, $n(x)$, from the shape function, $h(x)$

$$h(x) = h_0 + fx^2 + gx^4 \quad \text{(S4)}$$

$$n(x) = n_0 + \frac{2f}{\bar{H}} + \frac{12g}{\bar{H}}x^2 \quad \text{(S5)}$$

where x is the length along the x axis, and h_0, f, g are parameters of the expansion.

We next use this shape and protein distributions in the calculation of the total force F_{tot} acting on the membrane (Eqs.4-7). Using the Monge representation the curvature restoring force is of the form

$$F_{curv} = \frac{1}{2}\kappa\frac{(5 - 30h'^2)h''^3 + 20h'(1 + h'^2)h''h^{(3)} - 2(1 + h'^2)^2h^{(4)}}{(1 + h'^2)^{9/2}} \text{(S6)}$$

At steady state we look for a solution for h_0, f, g that obeys the vanishing of the total force, up to fourth order terms. To simplify matters we looked at the two separate cases of $\alpha > 0, A_{actin} = 0$, and $\alpha = 0, A_{actin} > 0$, and we kept the equations for h_0, f, g up to second order.

1.1 Adhesion-driven peak

When $\alpha > 0, A_{actin} = 0$, we obtain that

$$f_\alpha = \frac{-\bar{H}\left(2\left(\sigma - n_0\alpha\right) + \bar{H}^2 n_0^2 \kappa - 2Jn_0^2\right)}{16\left(\alpha - \bar{H}^2 n_0 \kappa + 2Jn_0\right)} \tag{S7}$$

and

$$g_\alpha = \frac{\bar{H}\gamma}{72(\kappa n_0 \bar{H}^2 - \alpha + 2Jn_0)} \tag{S8}$$

We find that these solutions behave as: $f < 0$ and $g > 0$, for strong adhesion (large α), which corresponds to a peak in the membrane shape and a similar peak in the membrane protein distribution (Eqs.S4,S5 and $\bar{H} < 0$). This is similar to the steady-state shape seen in the numerical simulations (Fig.2a). We see in Eqs.S7,S8 that the adhesion (α) competes with the elastic stiffness of the membrane that is induced by the membrane proteins (κ and J terms).

1.2 Actin-driven minimum

In a similar fashion the analytic derivation was performed for the case of $\alpha = 0, A_{actin} > 0$. The solutions we find are

$$f_{actin} = \frac{2\left(A_{actin} + \bar{H}\sigma\right) + \bar{H}^3 n_0^2 \kappa - 2\bar{H}Jn_0^2}{16\bar{H}^2 n_0 \kappa - 32Jn_0} \tag{S9}$$

and

$$g_{actin} = \frac{\bar{H}\gamma}{72\bar{H}\kappa n_0 - 144Jn_0} \tag{S10}$$

We find that these solutions behave as: $f > 0$ and $g < 0$, for strong actin protrusive force (large A_{actin}), which corresponds to a dip in the membrane shape and a similar dip in the membrane protein distribution (Eqs.S4,S5 and $\bar{H} < 0$). This is similar to the steady-state shape seen in the numerical simulations (Fig.2c). We see in Eqs.S9,S10 that the actin force (A_{actin}) competes with the membrane tension (σ) and the elastic stiffness of the membrane that is induced by the membrane proteins (κ and J terms).

2 Effect of the membrane curvature along the cell thickness

As shown in Fig.1b, a flat adhering cell has a relatively high membrane curvature along its thickness, of the order of $|H_0| \sim 1\mu\text{m}^{-1}$. The curvature term appearing in the free energy (Eq.1) which includes this contribution, is

$$\mathcal{F}_{curv} = \int \Big(\frac{1}{2}\kappa((H_0 + H) - \bar{H}n)^2 \Big) ds, \tag{S11}$$

Note that $H_0 < 0$ representing a convex shape.

When we do the variation on this energy, we find that the forces acting on the membrane are as given in Eqs.4-7, simply that the parameters get renormalized as: (i) The membrane tension is increased; $\sigma \to \sigma + \kappa H_0^2$, (ii) there is an increase in the effective adhesion of the membrane to the substrate, since the convex membrane protein ($\bar{H} < 0$) help stabilize the curvature in the thickness direction; $\alpha \to \alpha + \bar{H}H_0\kappa$.

Finally, there is an additional constant force due to this curvature in the thickness direction, which arises from the membrane tension terms, since there is now a constant term in the mean curvature $H + H_0$. This force that acts along the outer cell contour, points inwards, and is balanced in our model by the bulk modulus term in Eq.2. We can therefore simply absorb this additional force inside the constant parameter S_{target} for the equilibrium area of the cell.

Since the effective membrane tension and the adhesion strength are free parameters, these renormalizations do not alter qualitatively our results.

3 Effect of local restoring force on the actin polymerization rate

We wish to study the effect of making the polymerization rate A_{actin} locally dependent on the restoring force applied by the membrane. We use the phenomenological relation between polymerization rate and applied force that is used in [1], which is

$$A_{actin} = A_{actin,0} \left(1 - \left(\frac{F_{rest} n_0}{F_s n} \right)^w \right) \tag{S12}$$

where F_{rest} is the sum of the membrane restoring forces (Eqs.4-7), $A_{actin,0}$ is the bare polymerization rate without an opposing force, and w is some power

larger than 2. Note that the polymerization rate is related to the load force per filament, so is dependent on the local density n. If the membrane force is pointing outwards, it does not oppose the actin polymerization, which then attains its bare value $A_{actin,0}$.

In Fig.S1 we plot the membrane shape and membrane protein distribution for the case of actin force, with and without the local force dependence. We chose the parameters: $w = 4$, $F_s = 0.3 \cdot 10^{-3}$grμm^{-1}sec^{-2} and $A_{actin} = 0.015$grμm^{-1}sec^{-2}. We find that there is now a reduced rate of actin polymerization where the membrane is convex due to the stronger membrane restoring force, so that the positive feedback between the membrane shape and membrane protein distribution is weakened. On the other hand this is slightly, but not fully, compensated by the fact that exactly there the membrane load is being shared by more filaments (Eq. S12). The coalescence of the protrusions is less effective and therefore their number stays larger for longer times.

Figure S1: **Effects of local restoring force on rate of actin polymerization.** (a) Numerical simulations of the evolution of the membrane shape and membrane protein distribution, for the flat geometry, driven by actin alone. Black lines give the results without the effects of the local force, while red lines are including the effects of the local membrane restoring force. (b) The number of protrusions as a function of time for the two calculations shown in (a), with the same color code.

4 Movie legends

Supporting Movie 1 This is the simulation for the round cell driven purely by adhesion (Fig.2b). The top panel shows the membrane shape, while the bottom panel gives the concentration distribution of the membrane protein.

Supporting Movie 2 This is the simulation for the round cell driven purely by actin polymerization (Fig.2d). The top panel shows the membrane shape, while the bottom panel gives the concentration distribution of the membrane protein.

Supporting Movie 3 This is the simulation for the round cell driven purely by actin polymerization, with a polarized initial distribution of the

membrane proteins (Fig.2e). The top panel shows the membrane shape, while the bottom panel gives the concentration distribution of the membrane protein.

References

[1] Mogilner A, Rubinstein B (2010) Actin disassembly clock and membrane tension determine cell shape and turning: a mathematical model. J Phys: Condens Matter 22: 194118.
